# Seasonal Drought Timing Shapes Flowering Phenology Directly and Through Biotic Interactions

**DOI:** 10.1111/ele.70438

**Published:** 2026-07-19

**Authors:** Barel Tsafon, Or Gross, Niv DeMalach

**Affiliations:** ^1^ Institute of Plant Sciences and Genetics in Agriculture, Faculty of Agriculture Food and Environment The Hebrew University of Jerusalem Rehovot Israel

**Keywords:** annual plants, climate change, competition, drought timing, flowering, Mediterranean ecosystems, niche partitioning, phenology, phenotypic plasticity, seasonality

## Abstract

Flowering time underpins plant fitness, species coexistence, and ecosystem functioning. While global warming consistently advances flowering, the influence of water availability remains unclear. We hypothesized that this inconsistency reflects the overlooked timing of drought. In 200 experimental Mediterranean annual‐plant communities, we imposed autumn, winter, and spring dry periods and grew plants in monocultures and mixtures to disentangle physiological and competition‐mediated responses. Dry autumn and spring shortened flowering duration: dry autumn delayed onset, dry spring advanced termination. Some shifts were direct; others emerged through competition. A new community‐level index revealed greater phenological segregation in mixtures, showing that plasticity alone can generate niche separation under competition. Both dry autumn and spring tended to further enhance this segregation. Together, our results demonstrate that the seasonal timing of drought governs flowering responses through both direct physiological pathways and indirect biotic interactions, emphasizing rainfall seasonality as a key driver of ecological responses to climate change.

## Graphical abstract

The effects of drought on flowering depend on when drought occurs during the growing season. In experimental monocultures and mixtures, autumn drought delayed flowering onset whereas spring drought advanced flowering termination, with both reducing flowering duration through direct physiological effects and competition‐mediated interactions. A new community‐level index revealed greater phenological segregation in mixtures, showing that plasticity alone can generate niche separation under competition.

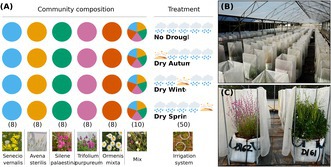

## 1 Introduction

Flowering phenology, the timing of flowering onset and termination, is a fundamental biological trait that shapes individual fitness and underpins key ecosystem functions and services (Kantsa et al. 2018; Taylor et al. 2019; Ren et al. 2024). The timing of major flowering events has broad public relevance with various cultural and economic consequences globally (Winkler and Brooks 2020; Graves et al. 2017). Moreover, since flowering time influences competition for pollinators and other resources, it is recognized as a critical component of niche partitioning, where temporal segregation across the growing season reduces competitive overlap and promotes species coexistence (Sherry et al. 2007).

There is mounting evidence that climate change is altering phenological patterns across ecosystems, with cascading consequences for community structure, plant–pollinator interactions, and ecosystem stability (Liu et al. 2025; Fitter and Fitter 2002; Vitasse et al. 2022; Høye et al. 2013). Hence, flowering phenology is often viewed as a direct indicator of ecosystem change (Alexander and Levine 2019; Cleland and Wolkovich 2024).

It is well established that increased temperature triggers earlier flowering phenology (Cook et al. 2012; Peñuelas and Filella 2001; Wolkovich et al. 2012; Williamson et al. 2025), but the effects of changes in precipitation patterns are unclear, with conflicting results (Knapp et al. 2024; Lu et al. 2023; Martén‐Rodríguez et al. 2025; Matthews and Mazer 2016). In seasonally dynamic ecosystems, such as Mediterranean systems, water availability is a limiting factor, and the impact of drought may depend strongly on its timing within the growing season (Ru et al. 2024; Gordo and Sanz 2010; Johansson et al. 2013; Reyer et al. 2013). It is now clear that climate change is causing these ecosystems to experience a shortening of the wet season, along with shifts in rainfall timing and distribution (Delworth et al. 2022; Vicente‐Serrano et al. 2025). However, so far, experimental studies of flowering phenology have focused on altering the total rainfall amount rather than its timing (Castillioni et al. 2022; Van Dyke and Kraft 2025).

The impact of climate change on plants is recognized as both direct, through physical changes, and indirect, through biotic interactions (Chu et al. 2016). Unlike temperature, the water available to a plant is influenced not only by abiotic factors but also through depletion caused by neighbouring individuals (Trautz et al. 2017). This makes interspecific competition an even more critical consideration, yet one that very few phenology experiments have addressed. The importance of this perspective is particularly evident in the case of flowering, with empirical work showing that temporal patterns shift as species diversity changes (Wolf et al. 2017). Ultimately, two key questions arise: first, how do plants adjust their flowering phenology in response to temporal changes in water availability, and second, can competitive interactions between species modify this effect?

**FIGURE 1:**
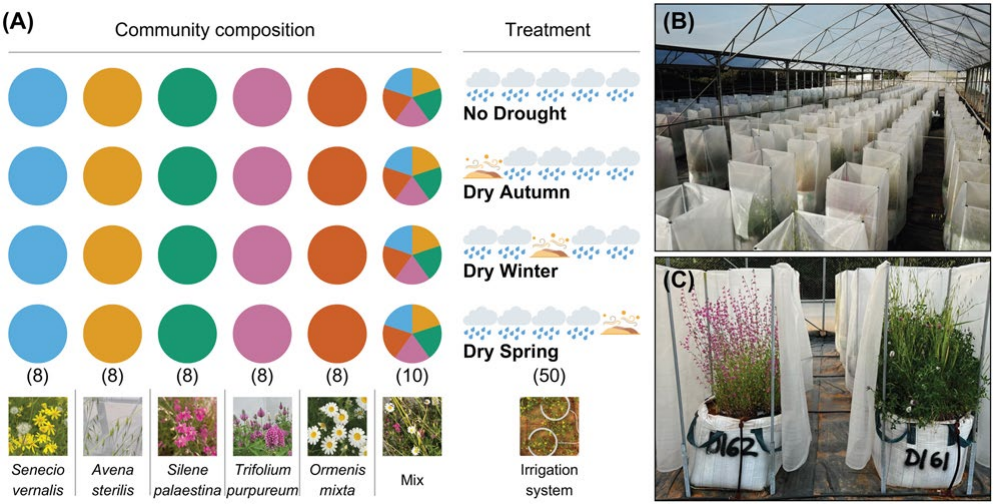
Overview of the experimental design. (A) Schematic illustration of the experimental treatments. Each circle represents one community; coloured circles correspond to monocultures of five focal species (eight replicates each), and the multicoloured pie chart represents mixed species communities (ten replicates). Treatments include a control (no drought) and three dry periods in autumn, winter, or spring. (B) Photograph of the greenhouse showing the full set of experimental units. (C) Example of two experimental communities: A *Silene palaestina* monoculture (left) and a five‐species mixture (right).

Here, we experimentally manipulated the timing of water availability in controlled settings to test its effects on species‐level flowering phenology and community‐level phenological overlap (Figure 1). We established annual plant communities subjected to a control and three dry periods (autumn, winter, and spring), each involving the same reduction in total water supply, thereby mimicking the shortening of the rainy season observed in Mediterranean systems worldwide (Vicente‐Serrano et al. 2025). Each species was grown in monocultures and mixed communities to disentangle direct physiological responses to treatments from effects mediated by interspecific interactions (hereafter, ‘competition’). In addition, we developed a new index to capture the degree of multi‐species niche partitioning at the community level.

Four hypotheses were tested: (1) Drought effects are both direct and mediated by species interactions; (2) Dry autumn delays flowering onset due to postponed germination, thereby reducing the flowering period; (3) Dry spring causes early ending of flowering (hereafter offset) due to early‐season termination, also reducing the flowering period; (4) Autumn‐ and spring‐droughts constrain phenological extremes, thereby reducing phenological niche partitioning and increasing overlap.

## 2 Materials and Methods

### 2.1 Establishment of the Experiment

The experiment started in October 2022 in a greenhouse located at the agricultural campus of the Hebrew University of Jerusalem in Rehovot, Israel (31.90°, 34.80°). The greenhouse had a polyethylene roof to prevent natural precipitation and net walls (20 × 20 cm mesh opening) that enabled maximum ventilation with temperatures close to ambient. The climate in the region is Mediterranean with mild rainy winter (∼560 mm y^−1^, falling mainly between October and April) and a dry, hot summer (Issaka et al. 2023).

The experimental communities mimicked the natural annual plant communities growing on the red sandy soil of Israel's coastal region (Hamra communities, (Issaka et al. 2023)). We chose five common species varying in phenology, functional group, and taxonomic group: (1) *Senecio vernalis* Waldst. & Kit. (Compositae), typically flowering from December to February. (2) *Avena sterilis* L. (Poaceae), flowering from March to May; (3) *Silene palaestina* Boiss. (Caryophyllaceae), flowering from March to April; (4) *Trifolium purpureum* Loisel. (Fabaceae), flowering from March to April; (5) *Ormenis mixta* (L.) Chevall. (Compositae), flowering from April to May. All seeds were collected in the spring of 2021 from nearby natural sites.

The experiment included 200 units; communities grown in industrial bags (0.7×0.7×0.7 m) spaced at least 0.8 m apart. Every bag was surrounded by a 50‐mesh net that rose 1 m above the soil surface to minimize dispersal. Hamra soil was collected from a depth of at least 1 m in the area surrounding the experimental site, to minimize the presence of a natural seed bank and ensure that only sown species emerged. It was fertilized with 5.26 g m^−2^ Osmocote Pro fertilizer (2 g N m^−2^) to compensate for the lack of organic matter at that depth of this nutrient‐poor soil.

Each species was grown in a monoculture or together with the other four species in mixture communities (Figure 1). Monocultures enable the quantification of the direct impact of treatments on any of the species, while the difference between mixtures and monocultures allows the estimation of the competition‐mediated effects. Based on the preliminary germination trials, we sowed seeds aiming for an initial density of 1000 seedlings per square metre (in mixture units: 200 seedlings per species).

On October 10th, we sowed seeds for most treatments just before the irrigation schedule began. In the dry autumn treatment, where irrigation was delayed, we sowed on November 16th to prevent pre‐irrigation seed loss and to align sowing with the start of water availability, as in all other irrigation treatments.

### 2.2 Experimental Schedule

To ensure that each community received a separate treatment, irrigation was applied through the NetBow drip irrigation system (by Netafim Ltd.). Two NetBow rings were installed in each bag (flow rate was 16 mm/h per bag). Irrigation events occurred once a week to mimic the natural cycles of wetting and drying. Each event lasted 1.5 h; thus, every week, the communities received 24 mm of water: 576 mm throughout the long season (control) and 456 mm in the drought treatments. Additionally, during the first three weeks after sowing, water was lightly added with a hose to enhance germination.

We created an irrigation schedule based on natural precipitation in the region, which historically lasts (on average) between mid‐October and the beginning of April. However, in the last 30 years, the number of rainy days has decreased by 10% (Drori et al. 2021). Therefore, we chose to examine a 20% reduction compared to the historical record. We designed the experiment such that all drought treatments would have the same number of irrigation events and amount of water to ensure that changes in phenology can be causally attributed to the timing of water stress within the growing season.

Each experimental unit was assigned to one of four drought types, reflecting that fewer rainy days can be expressed at different parts of the growing season (e.g., fewer early‐season rain events, an extended mid‐season dry spell, or fewer late‐season rain events). Accordingly, treatments concentrated the same number of ‘missing’ rain events into a five‐week dry period placed at different seasonal windows: (1) ‘no drought’– a 25‐week watering schedule with no imposed five‐week dry period, representing the historically common wet‐season pattern in the region; (2) ‘dry autumn’ – a shorter watering schedule of 20 weeks, starting 5 weeks after the control, representing years in which early‐season rainfall is reduced and the growing season begins later; (3) ‘dry winter’—a 25‐week schedule with a five‐week dry period in January (mid‐winter), representing years with a long rain‐free interval during the middle of the season despite a normal start and end; and (4) ‘dry spring’ – a shorter watering schedule of 20 weeks, starting as the control but ending five weeks earlier (before the beginning of spring), representing years in which late‐season rainfall is reduced and the wet season ends early. Experimental units were randomly assigned to create eight monoculture replicates per species per treatment and ten mixture replicates (Figure 1).

### 2.3 Sampling

At the beginning of the first and last months of irrigation, species abundances were estimated in each bag by counting individuals within a 40 cm × 40 cm quadrat placed at the centre. These counts provide the baseline species densities used to express weekly flowering counts as proportions for the phenology threshold (see below).

To describe phenology, we estimated each species' flowering window (onset to offset) and the degree of overlap among species within communities. We therefore surveyed each unit weekly and recorded, for each species, whether it was flowering (yes/no). A species was classified as flowering when ≥ 1% of its individuals had open flowers. We determined this by counting flowering individuals and converting counts to proportions using measured population densities, assessed across the entire surface area of the bag. Importantly, onset and offset were defined from this proportional time series, so they do not depend on the first or last flowering individual. Using a proportional threshold keeps the criterion comparable across monocultures and mixtures, standardizes detection across units, and reduces sensitivity to absolute population size and density differences among treatments.

During drought treatments, soil moisture was measured weekly in every bag using a soil moisture metre to assess treatment effects on soil water content (Figure S6). Measurements were taken at 10 cm depth, consistent with the shallow rooting of many annuals. Measurements were taken at fixed locations in the quadrat corners to minimize edge effects while preserving the integrity of the community centre.

### 2.4 Statistical Analysis

All analyses were conducted in R version 4.5.2. We used linear regression to test the effects of drought treatments (coded using dummy variables), interspecific competition (monoculture vs. mixture), and their interactions on several response variables. These included flowering onset, flowering offset, and length of the flowering period.

For drought effects, we report the contrasts between each drought treatment and the control, calculated separately for monocultures and mixtures. To account for multiple testing across these drought contrasts, we controlled the false discovery rate using the Benjamini–Hochberg procedure within monocultures and within mixtures for each response variable.

To quantify community‐level niche partitioning of the flowering season, we used the weekly flowering observations described above. For each community, we defined the flowering season as the number of weeks between the onset of the first flowering species and the offset of the last. We then calculated a standardized niche partitioning index for each community. The index ranges from 0 (no partitioning, complete overlap) to 1 (maximum partitioning, no overlap), and is defined by the following equation:

$$\text{Niche Partition}=1-\left(\sum_{i=1}^{n}\frac{i-1}{\;n-1\;}\;P_{i}\right)$$

where n is the number of species in the community, i is the number of flowering species, and $P_{i}$ is the proportion of the flowering season during which i species flower. The index assigns greater weight to weeks with more co‐flowering species, capturing the degree of phenological overlap. Subtracting this overlap score from one yields a measure of temporal niche partitioning at the community level.

The niche partitioning index was calculated per unit for the real mixed communities, reflecting the co‐flowering of species grown together. In contrast, monoculture communities were composed of single species grown in isolation, so we constructed simulated mixed communities by randomly selecting one monoculture replicate per species and combining their flowering dates to mimic the structure of a five‐species mixture, but without interspecific competition. While many such combinations are possible, most would not be statistically independent. To address this, we constructed eight non‐overlapping simulated mixed communities in each iteration, ensuring that each monoculture replicate appeared only once. This preserved independence across communities while maintaining the original sample size.

We ran a linear regression to estimate treatment effects, including the real and simulated mixed communities in each iteration. The procedure was repeated 100 times to minimize the stochastic effects of sampling from the monocultures. Parameter estimates from these iterations were averaged to robustly estimate treatment effects. Because p‐values cannot be averaged directly, we combined the iteration‐specific p‐values for each contrast using the Cauchy combination test (CCT), a standard approach for aggregating potentially dependent p‐values into a single valid combined p‐value (Liu and Xie 2020). Briefly, the CCT transforms each p‐value and sums the transformed values to form a test statistic with known null behaviour, without requiring independence among iterations. This yielded one integrated p‐value per contrast across the 100 iterations. Then, to account for multiple testing across drought‐treatment contrasts, we controlled for false discovery rate using the Benjamini–Hochberg procedure (Benjamini and Hochberg 1995), applied to the CCT‐integrated p‐values within monocultures and within mixtures.

## 3 Results

**FIGURE 2:**
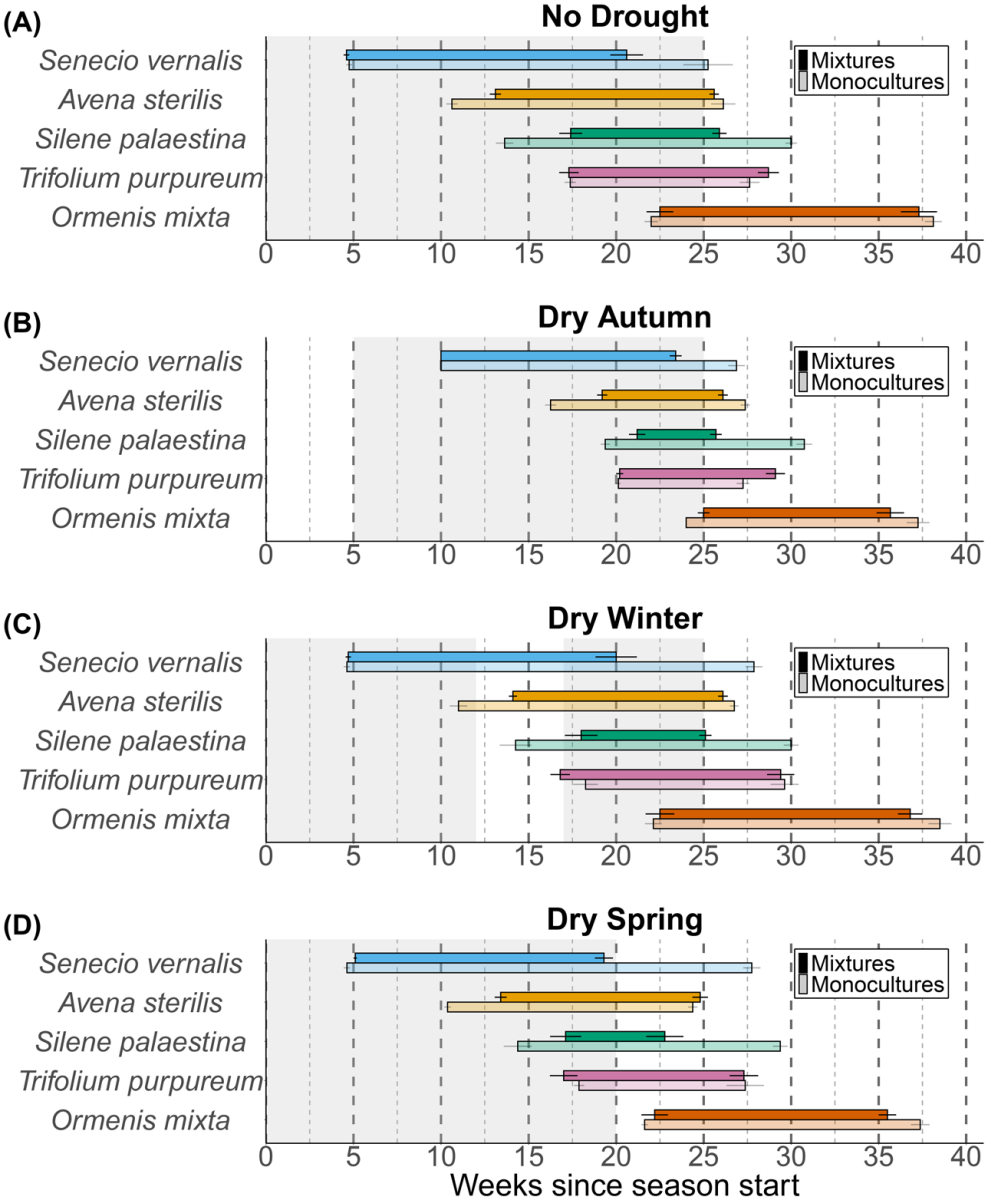
Drought and competition alter flowering phenology. Calendric flowering phenology of each species in monocultures and mixtures under four drought treatments (A–D; control and 3 different timings of a dry period). Each colour represents one species. Phenology is shown as the mean flowering window (onset to offset) for each species; error bars show ±SE for onset and offset estimates. Light and dark shades indicate monocultures and mixtures, respectively (mixtures contained all species with overall density matching the monocultures). The x‐axis shows weeks since the start of the season in mid‐October (Week 0 marks the onset of irrigation). Grey areas indicate periods with irrigation, while white areas indicate drought. *n* = 8 replicates per species × treatment in monocultures; *n* = 10 replicates per species × treatment in mixtures.

**FIGURE 3:**
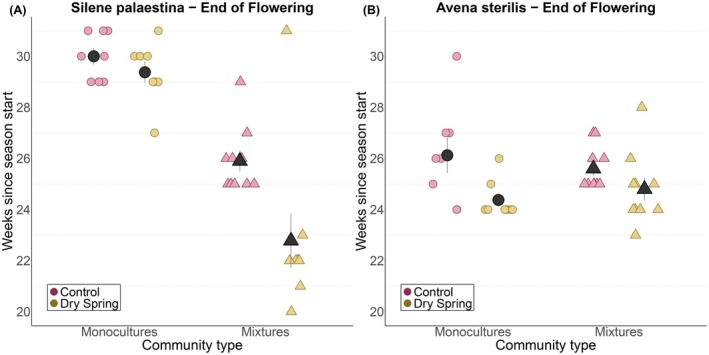
Direct and indirect effects of drought on flowering offset for (A) *Silene palaestina* and (B) *Avena sterilis* . The y‐axis is the timing of flowering offset (last flowering week; week 0 = start of irrigation). The x‐axis indicates the control vs. spring drought. Circles represent monocultures, and triangles represent mixtures. Black points show means; error bars show ±SE. Monoculture responses quantify the direct effects of drought (in the absence of competition), whereas mixture responses quantify the effects of drought in the presence of competition. *n* = 8 replicates per treatment in monocultures; *n* = 10 replicates per treatment in mixtures. BH‐adjusted *p* ‐values: *Silene*: Monoculture p=0.514; mixture p<0.001. *Avena*: Monoculture p=0.004; mixture p=0.211.

The phenological order of species in the experiment matched their natural flowering sequence. In all treatments, *Senecio* was the first to flower, with flowering onset within five weeks of irrigation initiation, followed by *Avena*, *Silene*, *Trifolium*, and finally *Ormenis*, which continued to flower well beyond the end of irrigation (Figure 2A). Interspecific competition shortened the flowering period of *Senecio*, *Silene*, and *Avena* by 3 to 8 weeks, as revealed by comparisons between their phenology in monocultures and mixtures (Figures 2A and S1; Table S1). Regarding the effects of drought timing, both dry autumn and spring shortened the flowering period, whereas dry winter had a negligible impact (Figure S1; Table S1). Dry autumn, characterized by a five‐week delay in irrigation, resulted in a species‐specific response of 2‐ to 5‐week delay in the onset of flowering across all species (Figure S2; Table S2). However, as this treatment did not affect offset (except in *Avena* monocultures), it led to a shorter overall flowering period (from the onset of the earlier species to the offset of the latest species) (Figures S2 and S3; Tables S2 and S3). Dry spring caused earlier flowering offset in the mid‐season species *Avena* and *Silene* (Figure 3; Table S3). In *Avena*, flowering offset advanced by 1.75 weeks in monocultures, consistent with a direct response to spring drought; however, in mixtures the estimated shift was less than 1 week (and statistically insignificant). In contrast, *Silene* showed no clear change in monocultures, but in mixtures it terminated flowering about 3 weeks earlier, implying that the impact of the drought was primarily mediated by species interactions (Figure 3; Table S3).

Interspecific competition increased the fraction of the flowering season with only one species flowering from 40% to 51%, thereby reducing the proportion of multispecies overlap (Figure S4). This was accompanied by a sharp decline in the proportion of time when all species flowered simultaneously, from 11% in monocultures to 3% in mixtures. The shift towards lower overlap was driven by shortened flowering durations in species that typically flower early or mid‐season, while the overall length of the flowering season remained unchanged (Figure 2A). Dry autumn further reduced the proportion of time during which multiple species flowered simultaneously (Figure S4). This was primarily due to a delayed onset in mid‐season species, while their offset remained unchanged (Figures 2B, S2 and S3; Tables S2 and S3). As a result, their overlap with the late‐flowering *Ormenis*, which also exhibited a delayed onset, was reduced. Finally, the dry spring reduced the number of days when four or five species flowered simultaneously (Figure S4), as mid‐season species, particularly *Silene* and *Avena*, terminated flowering earlier than under control conditions (Figure 3; Table S3).

**FIGURE 4:**
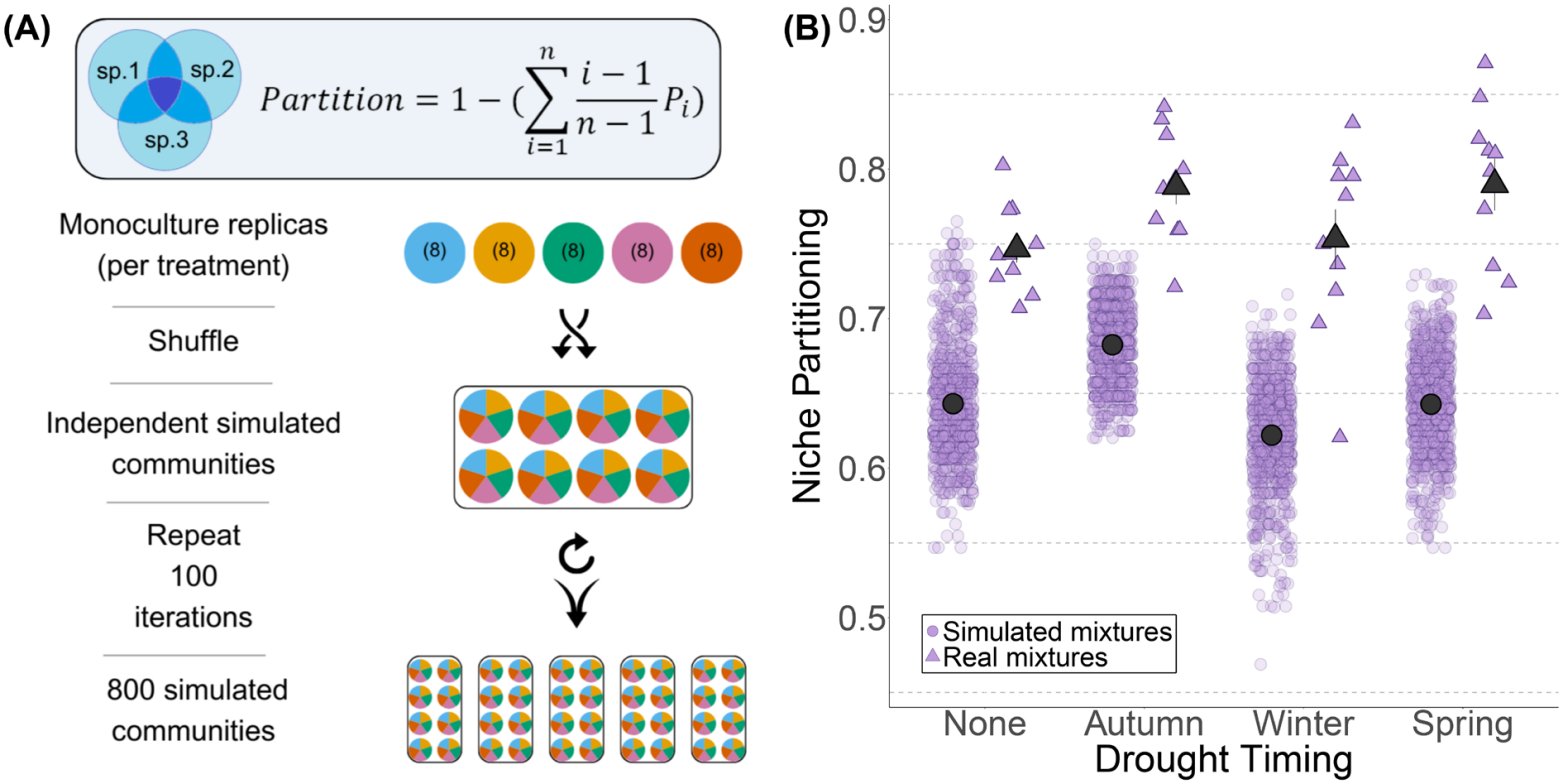
Competition and seasonal droughts alter phenological niche partitioning. (A) Overview of the niche partitioning index, quantifying the degree of temporal separation among species' flowering windows. The panel includes the equation used to calculate the index (see Methods) and a schematic representation of the simulation process for one treatment. (B) Effects of drought treatments (x‐axis) on the niche partitioning index (y‐axis). Triangles represent real mixed communities, whereas circles represent simulated mixed communities constructed from monoculture phenology (null expectation without species interactions). Error bars show standard errors of the mean. *n* = 10 real communities per treatment; *n* = 800 simulated communities per treatment. Pairwise drought‐treatment comparisons within real mixtures (BH‐adjusted p‐values): Control vs. dry autumn p=0.099, control vs. dry winter p=0.884, control vs. dry spring p=0.099, dry autumn vs. dry winter p=0.106, dry autumn vs. dry spring p=0.951, dry winter vs. dry spring p=0.106. Pairwise drought‐treatment comparisons within simulated mixtures (BH‐adjusted p‐values): Control vs. dry autumn p=0.141, control vs. dry winter p=0.406, control vs. dry spring p=0.997, dry autumn vs. dry winter p=0.038, dry autumn vs. dry spring p=0.141, dry winter vs. dry spring p=0.406. Real vs. simulated (regression model, unadjusted): p<0.001.

To quantify phenological niche partitioning at the community level, we developed a new index (Figure 4A; see Methods for details). Our index is based on the number of species flowering each week throughout the season (Figure S4). Weeks with more co‐flowering species indicate higher overlap, while weeks with fewer species indicate greater temporal segregation. The index is standardized between zero (maximum overlap, all species flower simultaneously) and one (maximum partitioning, each species flowers at a different time).

We compared niche partitioning in mixtures to expected values derived from monocultures. By randomly assembling the phenology data of monocultures into simulated mixed communities, we constructed a null model that excludes competition and enables the detection of direct drought effects (Figure 4A; see Methods for details). The niche partitioning was higher in the real mixtures compared to the simulated mixtures by ∼0.1, equivalent to a 10‐percentage point increase, across all treatments, indicating that interspecific competition enhanced the niche partitioning of flowering periods. Drought timing increased the community niche partitioning index by up to 0.04 (Figure 4B; although estimates were accompanied by substantial uncertainty Table S4). In simulated communities assembled from monocultures, only the autumn drought showed an increase in niche partitioning, and this estimate was highly imprecise (p=0.14). Yet, in the real mixed communities, both autumn and spring droughts were associated with increases in niche partitioning (p∼0.1; Table S4).

Calculating the community niche partitioning using the classical approach based on mean pair‐wise partitioning yielded similar results: the index of real mixtures was ∼0.1 higher than simulated mixtures, and dry spring further increased it (Figure S5; Tables S4 and S5).

## 4 Discussion

We found that the timing of drought shapes flowering phenology at the species level and phenological niche partitioning at the community level, with these effects arising through two mechanisms: directly in a species‐specific manner and indirectly through interspecific competition. A dry autumn delayed the flowering onset of early‐ and mid‐season species and appeared to reduce overlap with late‐season species. In contrast, a dry winter had no detectable effect on the onset or termination of flowering. Dry spring led to an earlier end of flowering, both directly (as seen in *Avena*) and indirectly through altered competitive dynamics (as in *Silene*), tending to increase phenological niche partitioning at the community level. Our findings also highlight the often‐overlooked role of competition‐induced phenotypic plasticity in shaping flowering phenology. Competition consistently shortened the flowering duration of most species, which contributed to increased phenological niche partitioning at the community level.

### 4.1 Competition and Phenological Niche Partitioning

In classical ecology, character displacement and limiting similarity describe an evolutionary process in which species diverge in their traits to reduce competition for shared resources (Macarthur and Levins 1967). This process often results in fixed differences in phenology, as observed in our monoculture treatments under control conditions, where species exhibited distinct flowering periods. However, our findings show that phenological niche partitioning can also arise within a single growing season through phenotypic plasticity in response to the immediate presence of other species. Indeed, previous work has demonstrated that plasticity, rather than adaptation, is the primary driver of flowering‐time variation along climatic gradients, highlighting its capacity to mediate rapid phenological shifts (Ramirez‐Parada et al. 2024). Our observation also aligns with recent theoretical work predicting that competition can induce plastic shifts in phenology (Levine et al. 2022; Rudolf 2019).

Plasticity‐driven niche partitioning has been well‐documented for below‐ground traits such as root allocation and nutrient uptake (Phoenix et al. 2020; Schiffers et al. 2011; Ashton et al. 2010), but it has rarely been studied in the context of flowering phenology. A notable exception is Wolf et al. (2017), who demonstrated that increased species richness led to greater variation in peak flowering time. This pattern was interpreted as evidence for niche partitioning driven by competition. Our results are consistent with their findings but differ in that we quantify partitioning based on the degree of overlap across the entire flowering period.

To date, two main approaches have been used to quantify phenological niche partitioning. One focuses on the variance or distance among peak flowering times (Williams 1995), while the other relies on flowering duration and temporal overlap, measured from onset to offset (Austin et al. 2024); both have advantages and disadvantages (Freitas and Bolmgren 2008).

Peak‐based metrics provide a clear summary of phenological timing differences and are useful for comparing shifts in central tendency, but peak dates alone may miss changes in onset and offset, and thus in the duration of co‐flowering.

Overlap‐based metrics address this limitation by directly quantifying co‐flowering, but they are most often implemented at the pairwise level (or summarized across species pairs), which can obscure higher‐order patterns when multiple species flower simultaneously. Several studies have also quantified phenological reassembly from a community‐level perspective using overlap‐related summaries such as the richness or composition of co‐flowering assemblages (Forrest et al. 2010; Theobald et al. 2017; Austin et al. 2024). Building on these approaches, we developed a simple scalar index that aggregates co‐flowering across all species at once. The close match between this index and the mean pairwise metric in our system (Figures 4B and S5) indicates that it is consistent with established overlap‐based measures while providing a compact community‐level summary that may become particularly informative as species richness and indirect interactions increase.

What drives plasticity‐induced niche partitioning? One possibility is that plants sense neighbouring individuals and adjust accordingly (Cahill et al. 2010). A simpler alternative is that plants respond to indirect environmental cues, such as declining water availability in mixtures (Wolf et al. 2017; Levine et al. 2024). The second explanation is supported by moisture patterns in our system (Figure S6), which were consistently lower in mixtures than all monocultures except for *Trifolium*. In *Trifolium* monocultures, moisture levels were similar to those in mixtures, which may explain why it was unaffected by interspecific competition.

Moreover, the phenological shifts observed under competition were consistent with each species' typical flowering schedule. The early‐season *Senecio* ceased flowering earlier under competition, the mid‐season species *Avena* showed earlier offset, and *Silene* had both earlier onset and offset. In contrast, *Ormenis*, which flowers late and develops deeper roots, was unaffected by competition, consistent with theoretical predictions that late‐season species are buffered from competition by rooting depth and reduced overlap (Levine et al. 2024).

These patterns are consistent with predominantly negative interactions in our system, as also shown in previous work (Issaka et al. 2023; Ayilara 2022); in other contexts, however, facilitation may buffer drought stress and thereby modify flowering timing and overlap patterns.

### 4.2 The Effects of Drought Timing

Many studies have examined how reduced precipitation influences flowering phenology, yet reported responses vary widely across systems and species (Knapp et al. 2024; Lu et al. 2023; Matthews and Mazer 2016; Van Dyke and Kraft 2025; Castillioni et al. 2022). Some studies document delayed flowering; others report earlier termination or shortened flowering duration, and the direction of response often depends on species identity and environmental context. Our results suggest that the position of drought within the growing season may contribute to these contrasting patterns.

In accordance with our second hypothesis, dry autumn, achieved by delaying the start of irrigation, resulted in later flowering onset across all species. This pattern suggests that a minimum plant size is required to trigger flowering (Wesselingh and de Jong 1995). Since biomass accumulation depends on time, delayed germination leads to delayed flowering. However, plants partially compensated for the delay: a 5‐week postponement of irrigation resulted in a delay of less than five weeks in the onset of flowering for most species. This indicates that flowering is regulated by a combination of cues, not by soil moisture alone, strengthening prior knowledge (Andrés and Coupland 2012).

We also hypothesized that a dry spring would lead to earlier flowering offset (Hypothesis 3), and this prediction was supported for two species, *Silene* and *Avena*. Interestingly, dry autumn affected only flowering onset, while dry spring affected only flowering offset. This indicates that the beginning and ending of the flowering period are not strictly linked, and that the total length of the flowering period is flexible rather than fixed (CaraDonna et al. 2014). In both cases, drought ultimately shortened the flowering period, in line with our expectations.

The dry autumn and spring extended the dry season at the leading and trailing edges of the wet season, respectively, whereas the dry winter treatment imposed only a brief drought interval after plants had reached maturity. This mid‐season pulse left both flowering onset and termination unchanged, indicating that established annuals can withstand short‐lived water deficits without shifting their reproductive phenology (Castillioni et al. 2022; Fenollosa et al. 2024).

In contrast with our prediction that drought would reduce phenological niche partitioning (Hypothesis 4), we found that it increased it. Our initial hypothesis was based on the assumption that dry autumn would primarily delay the flowering onset of early‐flowering species, and that dry spring would mainly trigger the early flowering offset of late‐flowering species. Instead, we observed that dry autumn delayed the onset across all species, not just early bloomers, though the extent of the delay varied. Specifically, mid‐season species showed a greater relative reduction in flowering duration. This led to an increased proportion of the flowering season dominated by single species, particularly early and late‐season species, thereby enhancing phenological niche partitioning.

Similarly, the dry spring treatment mainly affected mid‐season species. This again reduced the periods of co‐flowering and led to slightly higher levels of niche partitioning. Notably, while the competitive species *Avena* (Issaka et al. 2023) showed a primarily direct response, the drought‐induced shift in *Silene* occurred only under competitive conditions, demonstrating the role of species interaction in mediating drought response and aligning with our first hypothesis.

### 4.3 Limitations

As with any controlled experiment, our design trades some field realism for strong causal inference. The greenhouse setting with controlled irrigation reduced environmental variability and allowed us to isolate drought‐timing effects. In natural systems, the magnitude of phenological shifts and interaction strengths may also be altered by stochastic factors like temperature fluctuations, soil heterogeneity, and pollinator dynamics. Therefore, our results clarify how drought timing alone can shape phenology and temporal overlap, while recognizing that effect sizes may vary under more complex field conditions.

Phenology was surveyed weekly using a proportional flowering threshold. This resolution captures the multi‐week shifts central to our questions, but it may miss finer‐scale dynamics or short‐lived flowering pulses; therefore, very small differences (e.g., <1 week) should be interpreted cautiously. However, such differences are unlikely to alter the broader seasonal patterns analysed here.

Finally, the simulated ‘competition‐free’ mixtures were constructed by combining phenological data from monocultures. This approach represents a scenario in which species grow together without affecting each other directly or through higher‐order interactions, and assumes that species' phenological responses to drought are additive. However, monocultures can differ from mixtures in micro‐environmental conditions, which may influence phenology independently of interspecific interactions. Hence, differences between observed and simulated mixtures reflect neighbour context broadly (the net effect), rather than providing a detailed estimate of the interaction network.

## 5 Conclusion

We have shown that a dry autumn and a dry spring significantly affect flowering time, while a winter drought has little effect. This finding highlights the primary role of water availability timing in shaping phenology (Knapp et al. 2024). It is also relevant to the growing use of big data from herbarium records and citizen science in phenological research (Ahlstrand et al. 2025; Ramirez‐Parada et al. 2024), as the timing of rainfall events may serve as a better predictor of ecological patterns than annual precipitation totals (Poppenwimer et al. 2023), especially in Mediterranean ecosystems where shifts in rainfall timing are more reflective of observed climate change (Delworth et al. 2022; Vicente‐Serrano et al. 2025).

Lastly, our finding that drought effects are both direct and mediated by interspecific competition implies that responses cannot be fully understood from studies of single species. While the importance of indirect effects on community dynamics under climate change has been thoroughly recognized over the years (Adler et al. 2012; Van Dyke et al. 2022), phenology research has yet to adequately address these competition‐mediated effects. Understanding how biotic interactions shape phenological responses is therefore essential for predicting the future structure and resilience of ecological communities in times of global climate change.

## Author Contributions

All authors designed the experiment. B.T. and O.G. conducted the experiment. B.T. analysed the data. B.T. and N.D. wrote the manuscript with comments and suggestions from O.G.

## Funding

This work was supported by Israel Science Foundation (672/22, 2403/22), Women's League for Israel, School of Environmental Studies of the Hebrew University, Hebrew University Center for Sustainability, Ministry of Environmental Protection (22‐1‐18), Jewish National Fund.

## Conflicts of Interest

The authors declare no conflicts of interest.

## Supporting information


**Figure S1:** The number of flowering weeks (duration) of all species under the four drought treatments (first year). The y‐axis is the number of weeks with open flowers. The x‐axis is the treatments. Each species has its colour. Round points are for the species in monocultures, while triangular points are for the species in mixtures. The error bars are standard errors of means.
**Figure S2:** The number of weeks from the season start until the onset of flowering of all species under the four drought treatments (first year). The y‐axis is the number of weeks since the first irrigation. The x‐axis is the treatments. Each species has its own colour. Round points are for the species in monocultures, while triangular points are for the species in mixtures. The error bars are standard errors of means.
**Figure S3:** The number of weeks from the season start until the end of flowering of all species under the four drought treatments (first year). The y‐axis is the number of weeks since the first irrigation. The x‐axis is the treatments. Each species has its own colour. Round points are for the species in monocultures, while triangular points are for the species in mixtures. The error bars are standard errors of means.
**Figure S4:** The proportion of flowering weeks with different overlap intensities for each treatment. (A) monocultures and (B) mixtures.
**Figure S5:** The effect of treatments on the niche partitioning of whole communities (first year) as calculated by averaging the pairwise niche partitioning. The y‐axis is the averaged niche partitioning. The x‐axis is the drought treatments. Round points are for the aggregated monocultures communities, while triangular points are for the mixtures. The error bars are standard errors of means.
**Figure S6:** The soil moisture content of monocultures and mixtures during midwinter drought and under control (first year). The y‐axis is the soil moisture percentage at a depth of 10 cm. The x‐axis is the number of weeks that have passed since the drought started (0 means the soil was sampled on a week with irrigation). Each treatment has its shape. The error bars are standard errors of means.
**Table S1:** Linear regression coefficients and pairwise comparisons for flowering duration across species. Models were fit to each species separately. The upper part shows linear model coefficients, and the lower part shows pairwise contrasts, with Benjamini–Hochberg correction applied separately within each community. Units are the number of weeks between the start and end of flowering. Parentheses show the SE and *p*‐value for each coefficient or contrast.
**Table S2:** Linear regression coefficients and pairwise comparisons for flowering start across species. Models were fit to each species separately. The upper part shows linear model coefficients, and the lower part shows pairwise contrasts, with Benjamini–Hochberg correction applied separately within each community. Units are the number of weeks since the first irrigation. Parentheses show the SE and *p*‐value for each coefficient or contrast.
**Table S3:** Linear regression coefficients and pairwise comparisons for flowering end across species. Models were fit to each species separately. The upper part shows linear model coefficients, and the lower part shows pairwise contrasts, with Benjamini–Hochberg correction applied separately within each community. Units are the number of weeks since the first irrigation. Parentheses show the SE and *p*‐value for each coefficient or contrast.
**Table S4:** Linear regression coefficients and pairwise comparisons for niche partitioning across treatments. This table summarizes 100 regression analyses, each based on the same 40 mixtures and a different set of 32 independent simulated communities (‘Simulated’). The reported coefficients and *p*‐values are derived from these 100 models (see Methods for details). Units are niche partitioning values (0–1). The lower part shows pairwise contrasts, with Benjamini–Hochberg correction applied separately within each community to the integrated *p*‐values.
**Table S5:** Linear regression results for pairwise average niche partitioning across treatments. The model was fit for the 40 mixtures and one set of 32 independent communities. Units are the mean calculated niche partitioning value (0–1). The parentheses show the SE and *p*‐value for each predictor.

## Data Availability

All data and code supporting this study are openly available in figshare at https://doi.org/10.6084/m9.figshare.30383956 (Tsafon et al. 2025).
