# Supplementary material for: Seasonal Drought Timing Shapes Flowering Phenology Directly and Through Biotic Interactions
Source: Ecol Lett. 2026 Jul 19;29(7):e70438. doi: 10.1111/ele.70438 (PMC13382106; doi:10.1111/ele.70438)
Supplement: Supplementary file 1 — Figure S1: The number of flowering weeks (duration) of all species under the four drought treatments (first year). The y‐axis is the number of weeks with open flowers. The x‐axis is the treatments. Each species has its colour. Round points are for the species in monocultures, while triangular points are for the species in mixtures. The error bars are standard errors of means. Figure S2: The number of weeks from the season start until the onset of flowering of all species under the four drought treatments (first year). The y‐axis is the number of weeks since the first irrigation. The x‐axis is the treatments. Each species has its own colour. Round points are for the species in monocultures, while triangular points are for the species in mixtures. The error bars are standard errors of means. Figure S3: The number of weeks from the season start until the end of flowering of all species under the four drought treatments (first year). The y‐axis is the number of weeks since the first irrigation. The x‐axis is the treatments. Each species has its own colour. Round points are for the species in monocultures, while triangular points are for the species in mixtures. The error bars are standard errors of means. Figure S4: The proportion of flowering weeks with different overlap intensities for each treatment. (A) monocultures and (B) mixtures. Figure S5: The effect of treatments on the niche partitioning of whole communities (first year) as calculated by averaging the pairwise niche partitioning. The y‐axis is the averaged niche partitioning. The x‐axis is the drought treatments. Round points are for the aggregated monocultures communities, while triangular points are for the mixtures. The error bars are standard errors of means. Figure S6: The soil moisture content of monocultures and mixtures during midwinter drought and under control (first year). The y‐axis is the soil moisture percentage at a depth of 10 cm. The x‐axis is the number of weeks that have passed since the dr [file ELE-29-0-s001.pdf]

Supporting Information: Seasonal drought timing shapes flowering phenology directly and through biotic interactions

Barel Tsafon, Or Gross, Niv DeMalach

Institute of Plant Sciences and Genetics in Agriculture, Faculty of Agriculture, Food and Environment,
The Hebrew University of Jerusalem, Rehovot, Israel

Number of figures: 6
Number of tables: 5

*Correspondence: niv.demalach@mail.huji.ac.il

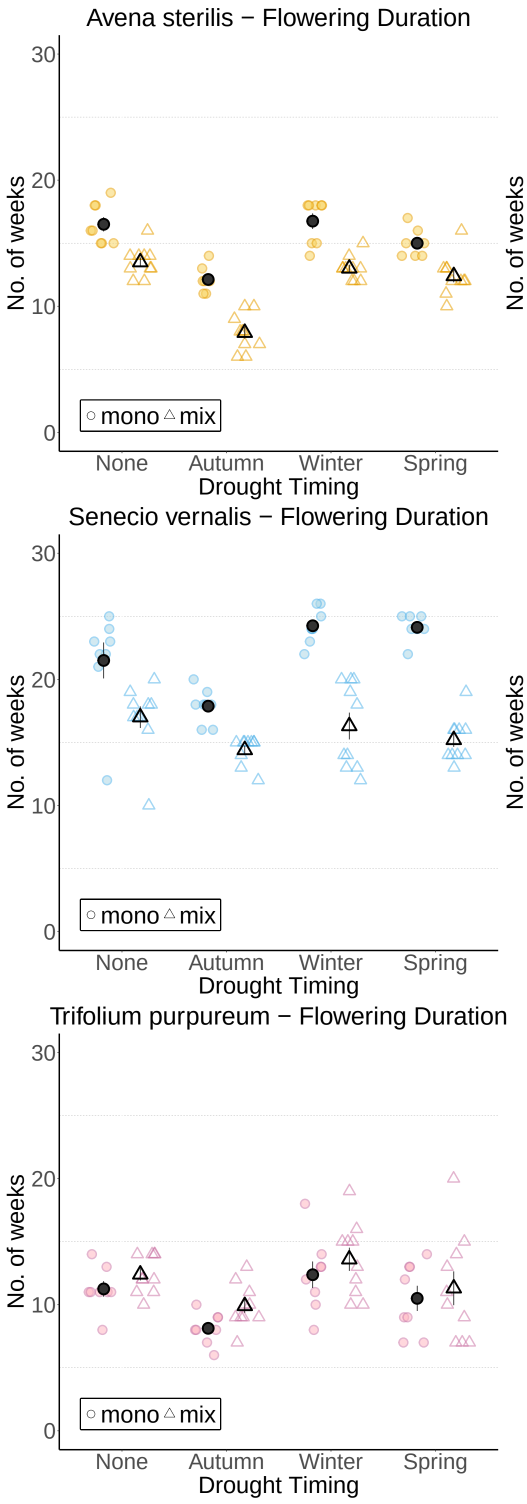

Figure 1: The number of flowering weeks (duration) of all species under the four drought treatments (first year). The y-axis is the number of weeks with open flowers. The x-axis is the treatments. Each species has its color. Round points are for the species in monocultures, while triangular points are for the species in mixtures. The error bars are standard errors of means.

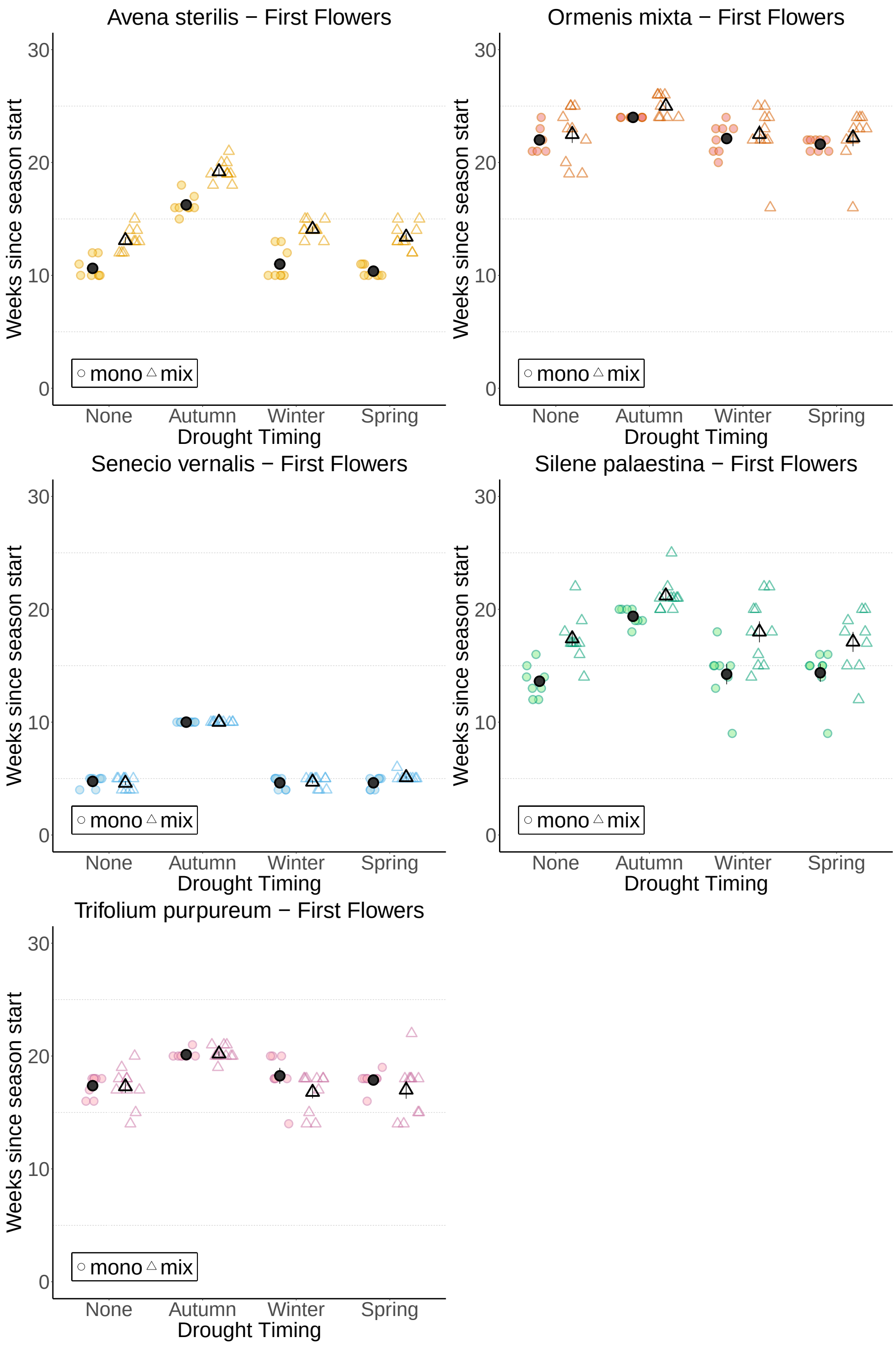

Figure 2: The number of weeks from the season start until the onset of flowering of all species under the four drought treatments (first year). The y-axis is the number of weeks since the first irrigation. The x-axis is the treatments. Each species has its own color. Round points are for the species in monocultures, while triangular points are for the species in mixtures. The error bars are standard errors of means.

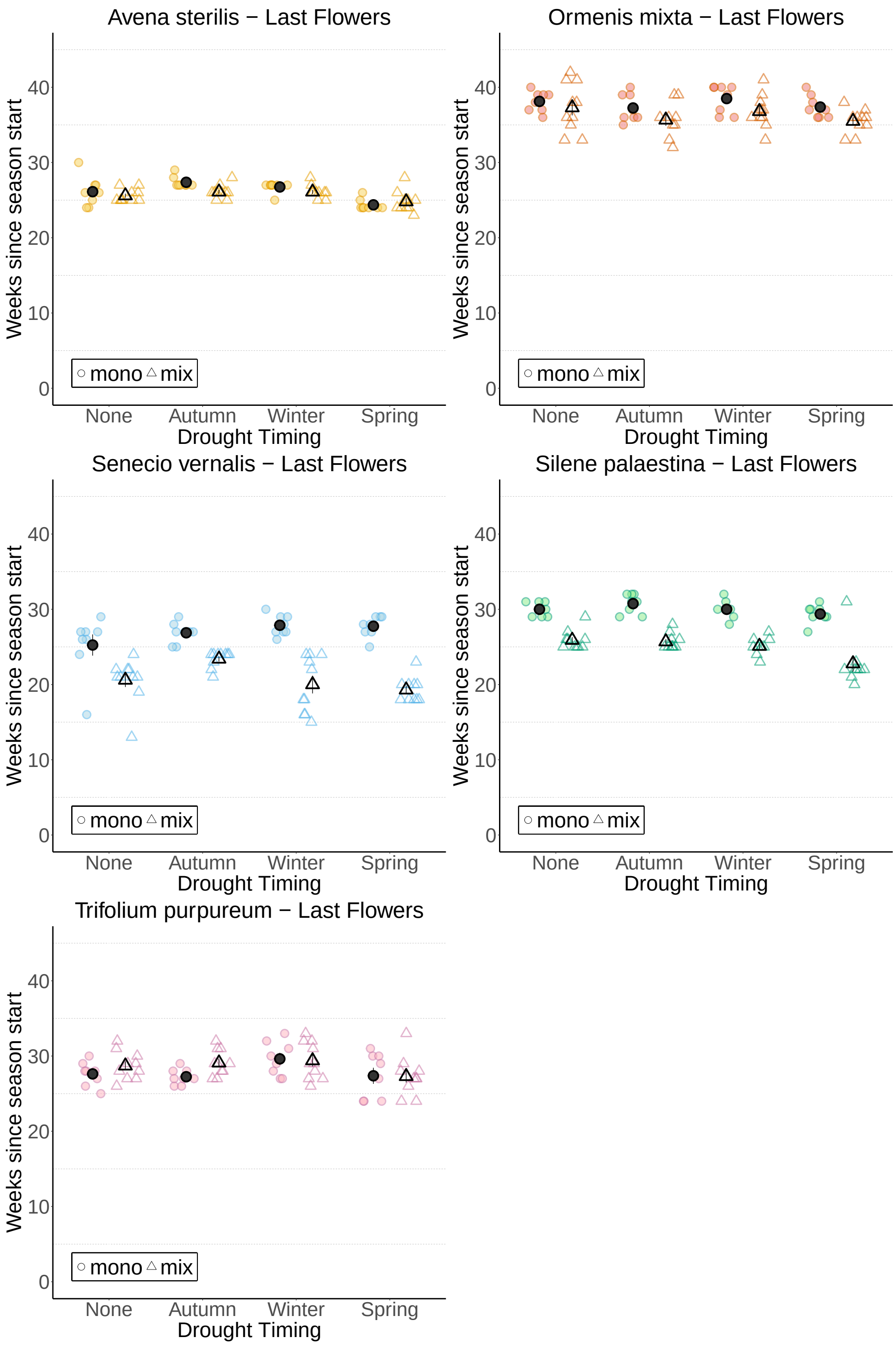

Figure 3: The number of weeks from the season start until the end of flowering of all species under the four drought treatments (first year). The y-axis is the number of weeks since the first irrigation. The x-axis is the treatments. Each species has its own color. Round points are for the species in monocultures, while triangular points are for the species in mixtures. The error bars are standard errors of means.

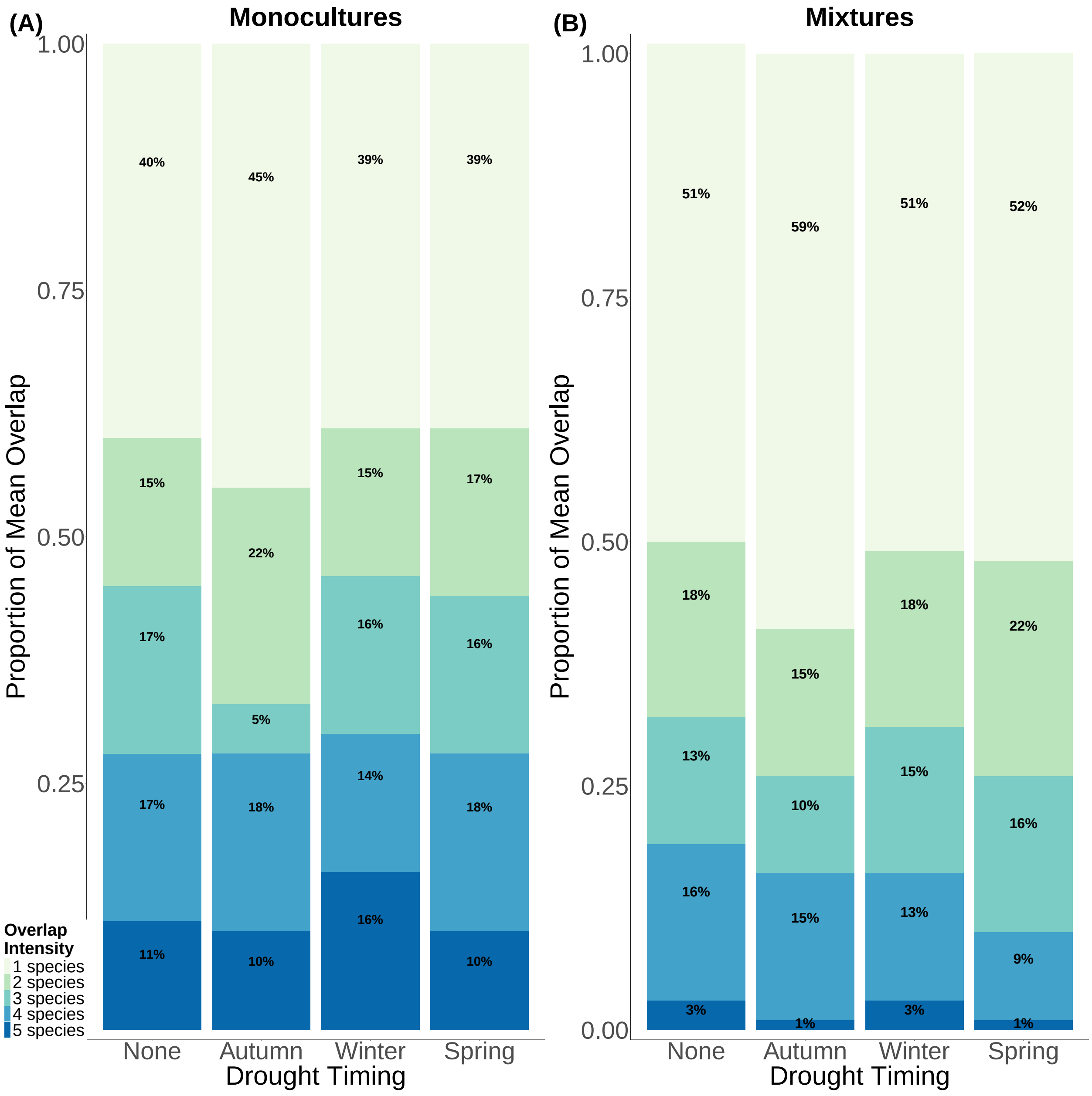

Figure 4: The proportion of flowering weeks with different overlap intensities for each treatment. (A) monocultures and (B) mixtures.

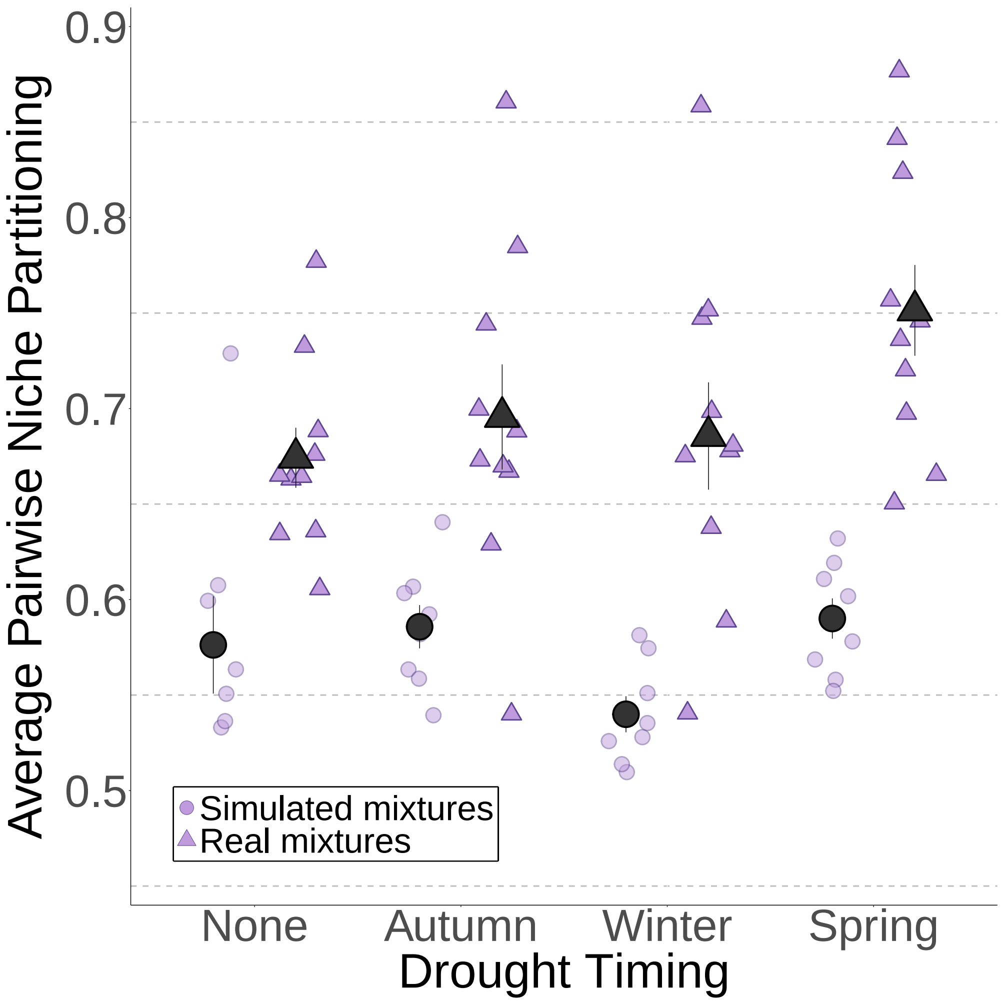

Figure 5: The effect of treatments on the niche partitioning of whole communities (first year) as calculated by averaging the pairwise niche partitioning. The y-axis is the averaged niche partitioning. The x-axis is the drought treatments. Round points are for the aggregated monocultures communities, while triangular points are for the mixtures. The error bars are standard errors of means.

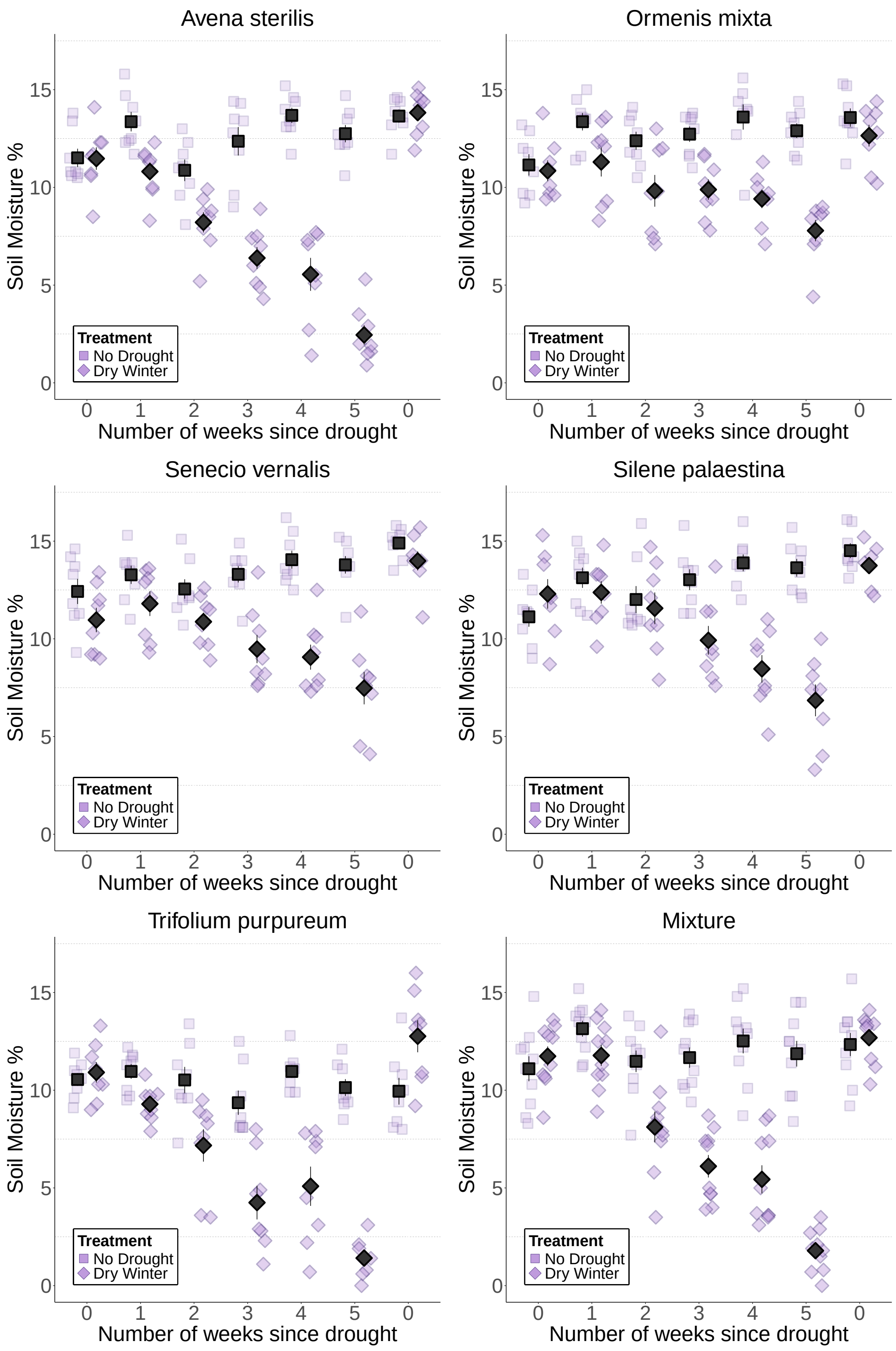

Figure 6: The soil moisture content of monocultures and mixtures during midwinter drought and under control (first year). The y-axis is the soil moisture percentage at a depth of 10cm. The x-axis is the number of weeks that have passed since the drought started (0 means the soil was sampled on a week with irrigation). Each treatment has its shape. The error bars are standard errors of means.

Table 1: Linear regression coefficients and pairwise comparisons for flowering duration across species. Models were fit to each species separately. The upper part shows linear model coefficients, and the lower part shows pairwise contrasts, with Benjamini–Hochberg correction applied separately within each community. Units are the number of weeks between the start and end of flowering. Parentheses show the SE and p-value for each coefficient or contrast.

Term	*Avena sterilis*	*Ormenis mixta*	*Senecio vernalis*	*Silene palaestina*	*Trifolium purpureum*
Intercept	16.500*** (0.476, p < 0.001)	17.125*** (1.059, p < 0.001)	21.500*** (0.831, p < 0.001)	17.375*** (0.975, p < 0.001)	11.250*** (0.923, p < 0.001
Dry Autumn	-4.375*** (0.674, p < 0.001)	-2.875 (1.498, p = 0.060)	-3.625** (1.175, p = 0.003)	-5.000*** (1.379, p < 0.001)	-3.125* (1.305, p = 0.020)
Dry Winter	0.250 (0.674, p = 0.712)	0.250 (1.498, p = 0.868)	2.750* (1.175, p = 0.022)	-0.625 (1.379, p = 0.652)	1.125 (1.305, p = 0.392)
Dry Spring	-1.500* (0.674, p = 0.029)	-0.375 (1.498, p = 0.803)	2.625* (1.175, p = 0.029)	-1.375 (1.379, p = 0.322)	-0.750 (1.305, p = 0.568)
Mixture	-3.000*** (0.639, p < 0.001)	-1.325 (1.421, p = 0.355)	-4.500*** (1.115, p < 0.001)	-7.875*** (1.308, p < 0.001)	1.150 (1.238, p = 0.356)
Dry Autumn:Mixture	-1.225 (0.904, p = 0.180)	-1.258 (2.035, p = 0.538)	1.025 (1.576, p = 0.518)	1.000 (1.850, p = 0.591)	0.625 (1.751, p = 0.722)
Dry Winter:Mixture	-0.750 (0.904, p = 0.410)	-0.750 (2.010, p = 0.710)	-3.450* (1.576, p = 0.032)	-0.775 (1.850, p = 0.677)	0.075 (1.751, p = 0.966)
Dry Spring:Mixture	0.400 (0.904, p = 0.660)	-1.125 (2.010, p = 0.578)	-4.425** (1.576, p = 0.007)	-1.458 (1.873, p = 0.439)	-0.350 (1.751, p = 0.842)
Mono: Control - Dry Autumn	4.375*** (0.674, p < 0.001)	2.875 (1.498, p = 0.179)	3.625** (1.175, p = 0.006)	5.000** (1.379, p = 0.003)	3.125 (1.305, p = 0.059)
Mono: Control - Dry Winter	-0.250 (0.674, p = 0.712)	-0.250 (1.498, p = 0.868)	-2.750* (1.175, p = 0.034)	0.625 (1.379, p = 0.652)	-1.125 (1.305, p = 0.470)
Mono: Control - Dry Spring	1.500* (0.674, p = 0.035)	0.375 (1.498, p = 0.868)	-2.625* (1.175, p = 0.035)	1.375 (1.379, p = 0.484)	0.750 (1.305, p = 0.568)
Mono: Dry Autumn - Dry Winter	-4.625*** (0.674, p < 0.001)	-3.125 (1.498, p = 0.179)	-6.375*** (1.175, p < 0.001)	-4.375** (1.379, p = 0.007)	-4.250* (1.305, p = 0.011)
Mono: Dry Autumn - Dry Spring	-2.875*** (0.674, p < 0.001)	-2.500 (1.498, p = 0.200)	-6.250*** (1.175, p < 0.001)	-3.625* (1.379, p = 0.021)	-2.375 (1.305, p = 0.147)
Mono: Dry Winter - Dry Spring	1.750* (0.674, p = 0.017)	0.625 (1.498, p = 0.868)	0.125 (1.175, p = 0.916)	0.750 (1.379, p = 0.652)	1.875 (1.305, p = 0.234)
Mix: Control - Dry Autumn	5.600*** (0.603, p < 0.001)	4.133* (1.377, p = 0.023)	2.600 (1.051, p = 0.096)	4.000* (1.233, p = 0.011)	2.500 (1.167, p = 0.106)
Mix: Control - Dry Winter	0.500 (0.603, p = 0.410)	0.500 (1.340, p = 0.710)	0.700 (1.051, p = 0.508)	1.400 (1.233, p = 0.315)	-1.200 (1.167, p = 0.350)
Mix: Control - Dry Spring	1.100 (0.603, p = 0.109)	1.500 (1.340, p = 0.401)	1.800 (1.051, p = 0.183)	2.833 (1.267, p = 0.078)	1.100 (1.167, p = 0.350)
Mix: Dry Autumn - Dry Winter	-5.100*** (0.603, p < 0.001)	-3.633* (1.377, p = 0.031)	-1.900 (1.051, p = 0.183)	-2.600 (1.233, p = 0.078)	-3.700* (1.167, p = 0.014)
Mix: Dry Autumn - Dry Spring	-4.500*** (0.603, p < 0.001)	-2.633 (1.377, p = 0.121)	-0.800 (1.051, p = 0.508)	-1.167 (1.267, p = 0.361)	-1.400 (1.167, p = 0.350)
Mix: Dry Winter - Dry Spring	0.600 (0.603, p = 0.388)	1.000 (1.340, p = 0.550)	1.100 (1.051, p = 0.449)	1.433 (1.267, p = 0.315)	2.300 (1.167, p = 0.106)
R-squared	0.815	0.278	0.727	0.742	0.292
No. of observations	72	71	72	71	72

Table 2: Linear regression coefficients and pairwise comparisons for flowering start across species. Models were fit to each species separately. The upper part shows linear model coefficients, and the lower part shows pairwise contrasts, with Benjamini–Hochberg correction applied separately within each community. Units are the number of weeks since the first irrigation. Parentheses show the SE and p-value for each coefficient or contrast.

Term	***Avena sterilis***	***Ormenis mixta***	***Senecio vernalis***	***Silene palaestina***	***Trifolium purpureum***
Intercept	10.625*** (0.340, p < 0.001)	22.000*** (0.623, p < 0.001)	4.750*** (0.144, p < 0.001)	13.625*** (0.761, p < 0.001)	17.375*** (0.549, p < 0.001)
Dry Autumn	5.625*** (0.480, p < 0.001)	2.000* (0.880, p = 0.027)	5.250*** (0.204, p < 0.001)	5.750*** (1.076, p < 0.001)	2.750*** (0.777, p < 0.001)
Dry Winter	0.375 (0.480, p = 0.438)	0.125 (0.880, p = 0.888)	-0.125 (0.204, p = 0.542)	0.625 (1.076, p = 0.564)	0.875 (0.777, p = 0.264)
Dry Spring	-0.250 (0.480, p = 0.604)	-0.375 (0.880, p = 0.672)	-0.125 (0.204, p = 0.542)	0.750 (1.076, p = 0.489)	0.500 (0.777, p = 0.522)
Mixture	2.475*** (0.456, p < 0.001)	0.500 (0.835, p = 0.552)	-0.150 (0.193, p = 0.441)	3.775*** (1.021, p < 0.001)	-0.075 (0.737, p = 0.919)
Dry Autumn:Mixture	0.475 (0.644, p = 0.464)	0.500 (1.196, p = 0.677)	0.150 (0.274, p = 0.585)	-1.950 (1.444, p = 0.182)	0.150 (1.042, p = 0.886)
Dry Winter:Mixture	0.625 (0.644, p = 0.336)	-0.125 (1.181, p = 0.916)	0.225 (0.274, p = 0.414)	-0.025 (1.444, p = 0.986)	-1.375 (1.042, p = 0.192)
Dry Spring:Mixture	0.550 (0.644, p = 0.397)	0.075 (1.181, p = 0.950)	0.625* (0.274, p = 0.026)	-1.039 (1.462, p = 0.480)	-0.800 (1.042, p = 0.445)
Mono: Control - Dry Autumn	-5.625*** (0.480, p < 0.001)	-2.000 (0.880, p = 0.074)	-5.250*** (0.204, p < 0.001)	-5.750*** (1.076, p < 0.001)	-2.750** (0.777, p = 0.004)
Mono: Control - Dry Winter	-0.375 (0.480, p = 0.525)	-0.125 (0.880, p = 0.888)	0.125 (0.204, p = 0.651)	-0.625 (1.076, p = 0.676)	-0.875 (0.777, p = 0.396)
Mono: Control - Dry Spring	0.250 (0.480, p = 0.604)	0.375 (0.880, p = 0.806)	0.125 (0.204, p = 0.651)	-0.750 (1.076, p = 0.676)	-0.500 (0.777, p = 0.626)
Mono: Dry Autumn - Dry Winter	5.250*** (0.480, p < 0.001)	1.875 (0.880, p = 0.074)	5.375*** (0.204, p < 0.001)	5.125*** (1.076, p < 0.001)	1.875* (0.777, p = 0.037)
Mono: Dry Autumn - Dry Spring	5.875*** (0.480, p < 0.001)	2.375 (0.880, p = 0.054)	5.375*** (0.204, p < 0.001)	5.000*** (1.076, p < 0.001)	2.250* (0.777, p = 0.015)
Mono: Dry Winter - Dry Spring	0.625 (0.480, p = 0.297)	0.500 (0.880, p = 0.806)	-0.000 (0.204, p = 1.000)	-0.125 (1.076, p = 0.908)	0.375 (0.777, p = 0.631)
Mix: Control - Dry Autumn	-6.100*** (0.430, p < 0.001)	-2.500** (0.809, p = 0.006)	-5.400*** (0.182, p < 0.001)	-3.800*** (0.963, p < 0.001)	-2.900*** (0.695, p < 0.001)
Mix: Control - Dry Winter	-1.000* (0.430, p = 0.035)	0.000 (0.788, p = 1.000)	-0.100 (0.182, p = 0.585)	-0.600 (0.963, p = 0.643)	0.500 (0.695, p = 0.711)
Mix: Control - Dry Spring	-0.300 (0.430, p = 0.487)	0.300 (0.788, p = 0.845)	-0.500* (0.182, p = 0.012)	0.289 (0.989, p = 0.771)	0.300 (0.695, p = 0.774)
Mix: Dry Autumn - Dry Winter	5.100*** (0.430, p < 0.001)	2.500** (0.809, p = 0.006)	5.300*** (0.182, p < 0.001)	3.200** (0.963, p = 0.003)	3.400*** (0.695, p < 0.001)
Mix: Dry Autumn - Dry Spring	5.800*** (0.430, p < 0.001)	2.800** (0.809, p = 0.006)	4.900*** (0.182, p < 0.001)	4.089*** (0.989, p < 0.001)	3.200*** (0.695, p < 0.001)
Mix: Dry Winter - Dry Spring	0.700 (0.430, p = 0.130)	0.300 (0.788, p = 0.845)	-0.400* (0.182, p = 0.038)	0.889 (0.989, p = 0.558)	-0.200 (0.695, p = 0.774)
R-squared	0.908	0.291	0.972	0.601	0.431
No. of observations	72	71	72	71	72

Table 3: Linear regression coefficients and pairwise comparisons for flowering end across species. Models were fit to each species separately. The upper part shows linear model coefficients, and the lower part shows pairwise contrasts, with Benjamini–Hochberg correction applied separately within each community. Units are the number of weeks since the first irrigation. Parentheses show the SE and p-value for each coefficient or contrast.

Term	***Avena sterilis***	***Ormenis mixta***	***Senecio vernalis***	***Silene palaestina***	***Trifolium purpureum***
Intercept	26.125*** (0.385, $p < 0.001$)	38.125*** (0.749, $p < 0.001$)	25.250*** (0.872, $p < 0.001$)	30.000*** (0.554, $p < 0.001$)	27.625*** (0.764, $p < 0.001$
Dry Autumn	1.250* (0.545, p = 0.025)	-0.875 (1.059, p = 0.412)	1.625 (1.234, p = 0.192)	0.750 (0.784, p = 0.342)	-0.375 (1.080, p = 0.730)
Dry Winter	0.625 (0.545, p = 0.255)	0.375 (1.059, p = 0.724)	2.625* (1.234, p = 0.037)	-0.000 (0.784, p = 1.000)	2.000 (1.080, p = 0.069)
Dry Spring	-1.750** (0.545, p = 0.002)	-0.750 (1.059, p = 0.481)	2.500* (1.234, p = 0.047)	-0.625 (0.784, p = 0.428)	-0.250 (1.080, p = 0.818)
Mixture	-0.525 (0.517, p = 0.313)	-0.825 (1.004, p = 0.415)	-4.650*** (1.170, $p < 0.001$)	-4.100*** (0.744, $p < 0.001$)	1.075 (1.025, p = 0.298)
Dry Autumn:Mixture	-0.750 (0.731, p = 0.309)	-0.758 (1.438, p = 0.600)	1.175 (1.655, p = 0.480)	-0.950 (1.052, p = 0.370)	0.775 (1.449, p = 0.595)
Dry Winter:Mixture	-0.125 (0.731, p = 0.865)	-0.875 (1.420, p = 0.540)	-3.225 (1.655, p = 0.056)	-0.800 (1.052, p = 0.450)	-1.300 (1.449, p = 0.373)
Dry Spring:Mixture	0.950 (0.731, p = 0.198)	-1.050 (1.420, p = 0.463)	-3.800* (1.655, p = 0.025)	-2.497* (1.065, p = 0.022)	-1.150 (1.449, p = 0.430)
Mono: Control - Dry Autumn	-1.250* (0.545, p = 0.038)	0.875 (1.059, p = 0.722)	-1.625 (1.234, p = 0.385)	-0.750 (0.784, p = 0.514)	0.375 (1.080, p = 0.908)
Mono: Control - Dry Winter	-0.625 (0.545, p = 0.255)	-0.375 (1.059, p = 0.869)	-2.625 (1.234, p = 0.141)	0.000 (0.784, p = 1.000)	-2.000 (1.080, p = 0.137)
Mono: Control - Dry Spring	1.750** (0.545, p = 0.004)	0.750 (1.059, p = 0.722)	-2.500 (1.234, p = 0.141)	0.625 (0.784, p = 0.514)	0.250 (1.080, p = 0.908)
Mono: Dry Autumn - Dry Winter	0.625 (0.545, p = 0.255)	-1.250 (1.059, p = 0.722)	-1.000 (1.234, p = 0.577)	0.750 (0.784, p = 0.514)	-2.375 (1.080, p = 0.124)
Mono: Dry Autumn - Dry Spring	3.000*** (0.545, $p < 0.001$)	-0.125 (1.059, p = 0.906)	-0.875 (1.234, p = 0.577)	1.375 (0.784, p = 0.506)	-0.125 (1.080, p = 0.908)
Mono: Dry Winter - Dry Spring	2.375*** (0.545, $p < 0.001$)	1.125 (1.059, p = 0.722)	0.125 (1.234, p = 0.920)	0.625 (0.784, p = 0.514)	2.250 (1.080, p = 0.124)
Mix: Control - Dry Autumn	-0.500 (0.487, p = 0.370)	1.633 (0.973, p = 0.294)	-2.800* (1.103, p = 0.027)	0.200 (0.701, p = 0.776)	-0.400 (0.966, p = 0.757)
Mix: Control - Dry Winter	-0.500 (0.487, p = 0.370)	0.500 (0.947, p = 0.719)	0.600 (1.103, p = 0.589)	0.800 (0.701, p = 0.387)	-0.700 (0.966, p = 0.707)
Mix: Control - Dry Spring	0.800 (0.487, p = 0.211)	1.800 (0.947, p = 0.294)	1.300 (1.103, p = 0.365)	3.122*** (0.720, $p < 0.001$)	1.400 (0.966, p = 0.304)
Mix: Dry Autumn - Dry Winter	-0.000 (0.487, p = 1.000)	-1.133 (0.973, p = 0.373)	3.400** (1.103, p = 0.009)	0.600 (0.701, p = 0.474)	-0.300 (0.966, p = 0.757)
Mix: Dry Autumn - Dry Spring	1.300* (0.487, p = 0.029)	0.167 (0.973, p = 0.865)	4.100** (1.103, p = 0.003)	2.922*** (0.720, $p < 0.001$)	1.800 (0.966, p = 0.201)
Mix: Dry Winter - Dry Spring	1.300* (0.487, p = 0.029)	1.300 (0.947, p = 0.349)	0.700 (1.103, p = 0.589)	2.322** (0.720, p = 0.004)	2.100 (0.966, p = 0.201)
R-squared	0.424	0.198	0.671	0.772	0.175
No. of observations	72	71	72	71	72

Table 4: Linear regression coefficients and pairwise comparisons for niche partitioning across treatments. This table summarizes 100 regression analyses, each based on the same 40 mixtures and a different set of 32 independent simulated communities ("Simulated"). The reported coefficients and p-values are derived from these 100 models (see Methods for details). Units are niche partitioning values (0–1). The lower part shows pairwise contrasts, with Benjamini–Hochberg correction applied separately within each community to the integrated p-values.

Term	***Whole communities***
(Intercept)	0.747*** ($p < 0.001$)
Dry Autumn	0.042* ($p = 0.033$)
Dry Winter	0.007 ($p = 0.737$)
Dry Spring	0.043* ($p = 0.029$)
Simulated	-0.104*** ($p < 0.001$)
Dry Autumn:Simulated	-0.002 ($p = 0.941$)
Dry Winter:Simulated	-0.028 ($p = 0.343$)
Dry Spring:Simulated	-0.043 ($p = 0.138$)
Mix: Control - Dry Autumn	-0.042 ($p = 0.100$)
Mix: Control - Dry Winter	-0.007 ($p = 0.885$)
Mix: Control - Dry Spring	-0.043 ($p = 0.100$)
Mix: Dry Autumn - Dry Winter	0.035 ($p = 0.107$)
Mix: Dry Autumn - Dry Spring	-0.001 ($p = 0.951$)
Mix: Dry Winter - Dry Spring	-0.036 ($p = 0.107$)
Simulated: Control - Dry Autumn	-0.039 ($p = 0.142$)
Simulated: Control - Dry Winter	0.021 ($p = 0.434$)
Simulated: Control - Dry Spring	0.001 ($p = 0.977$)
Simulated: Dry Autumn - Dry Winter	0.061* ($p = 0.038$)
Simulated: Dry Autumn - Dry Spring	0.041 ($p = 0.142$)
Simulated: Dry Winter - Dry Spring	-0.020 ($p = 0.434$)

Table 5: Linear regression results for pairwise average niche partitioning across treatments. The model was fit for the 40 mixtures and one set of 32 independent communities. Units are the mean calculated niche partitioning value (0–1). The parentheses show the SE and p-value for each predictor.

Term	***Pairwise***
(Intercept)	0.674*** (0.020, $p < 0.001$)
Dry Autumn	0.021 (0.029, $p = 0.463$)
Dry Winter	0.011 (0.029, $p = 0.695$)
Dry Spring	0.077** (0.029, $p = 0.010$)
Simulated	-0.098** (0.031, $p = 0.002$)
Dry Autumn:Simulated	-0.012 (0.043, $p = 0.786$)
Dry Winter:Simulated	-0.048 (0.043, $p = 0.276$)
Dry Spring:Simulated	-0.065 (0.043, $p = 0.140$)
R-squared	0.562
No. of observations	72
